# The effect of camicinal (GSK962040), a motilin agonist, on gastric emptying and glucose absorption in feed-intolerant critically ill patients: a randomized, blinded, placebo-controlled, clinical trial

**DOI:** 10.1186/s13054-016-1420-4

**Published:** 2016-08-01

**Authors:** Marianne J. Chapman, Adam M. Deane, Stephanie L. O’Connor, Nam Q. Nguyen, Robert J. L. Fraser, Duncan B. Richards, Kimberley E. Hacquoil, Lakshmi S. Vasist Johnson, Matthew E. Barton, George E. Dukes

**Affiliations:** 1 Department of Critical Care Services, Royal Adelaide Hospital, North Terrace, Adelaide, Australia; 2 Discipline of Acute Care Medicine, University of Adelaide, Adelaide, Australia; 3 Department of Gastroenterology and Hepatology, Royal Adelaide Hospital, Adelaide, Australia; 4 Discipline of Medicine, University of Adelaide, Adelaide, Australia; 5 Department of Gastroenterology and Hepatology, Flinders Medical Centre, Adelaide, Australia; 6 GlaxoSmithKline, Stevenage, UK; 7 GlaxoSmithKline, Research Triangle Park, Durham, USA

**Keywords:** Critical illness, Enteral nutrition, Absorption, Gastric emptying, Motilin agonist, Camicinal

## Abstract

**Background:**

The promotility agents currently available to treat gastroparesis and feed intolerance in the critically ill are limited by adverse effects. The aim of this study was to assess the pharmacodynamic effects and pharmacokinetics of single doses of the novel gastric promotility agent motilin agonist camicinal (GSK962040) in critically ill feed-intolerant patients.

**Methods:**

A prospective, randomized, double-blind, parallel-group, placebo-controlled, study was performed in mechanically ventilated feed-intolerant patients [median age 55 (19–84), 73 % male, APACHE II score 18 (5–37) with a gastric residual volume ≥200 mL]. Gastric emptying and glucose absorption were measured both pre- and post-treatment after intragastric administration of 50 mg (*n* = 15) camicinal and placebo (*n* = 8) using the ^13^C-octanoic acid breath test (BTt_1/2_), acetaminophen concentrations, and 3-O-methyl glucose concentrations respectively.

**Results:**

Following 50 mg enteral camicinal, there was a trend to accelerated gastric emptying [adjusted geometric means: pre-treatment BTt_1/2_ 117 minutes vs. post- treatment 76 minutes; 95 % confidence intervals (CI; 0.39, 1.08) and increased glucose absorption (AUC_240min_ pre-treatment: 28.63 mmol.min/L vs. post-treatment: 71.63 mmol.min/L; 95 % CI (1.68, 3.72)]. When two patients who did not have detectable plasma concentrations of camicinal were excluded from analysis, camicinal accelerated gastric emptying (adjusted geometric means: pre-treatment BTt_1/2_ 121 minutes vs. post-treatment 65 minutes 95 % CI (0.32, 0.91) and increased glucose absorption (AUC_240min_ pre-treatment: 33.04 mmol.min/L vs. post-treatment: 74.59 mmol.min/L; 95 % CI (1.478, 3.449). In those patients receiving placebo gastric emptying was similar pre- and post-treatment.

**Conclusions:**

When absorbed, a single enteral dose of camicinal (50 mg) accelerates gastric emptying and increases glucose absorption in feed-intolerant critically ill patients.

**Trial registration:**

The study protocol was registered with the US NIH clinicaltrials.gov on 23 December 2009 (Identifier NCT01039805).

## Background

Enteral nutrient delivery is frequently inadequate in the critically ill [1]. Poor nutrition results in impaired immune function, prolonged ventilator dependence, increased infectious complications, and overall poorer outcomes when compared to those that receive adequate nutrition [2]. Gastric emptying (GE) is slowed in up to 50–60 % of mechanically ventilated patients, resulting in an inability to deliver adequate nutrition by the gastric route [3]. This is manifest as increased gastric residual volumes (GRV), or regurgitation and vomiting, which can lead to aspiration of gastric contents, resulting in respiratory compromise and ventilator-associated pneumonia [3].

Enhancing gastric emptying by the administration of gastrokinetic agents is a recommended strategy for optimizing delivery and minimizing the risks of enteral nutrition (EN) [4]. Gastrokinetic drugs such as erythromycin and metoclopramide have been shown to acutely accelerate GE, reduce GRVs, and increase delivery of calories via the gastric route [5–12].

Metoclopramide is an antagonist of central and peripheral dopamine receptors and has been reported to be somewhat useful as a gastrokinetic drug [7], although less effective than erythromycin [10–12]. Concerns regarding extrapyramidal side effects have also been reported [13]. Erythromycin is a macrolide antibiotic and motilin receptor agonist, and while it is a potent gastrokinetic drug, there is the potential risk of QT prolongation and cardiac arrhythmias [14]. Additionally, there is concern that widespread use of this antibiotic in non-infected patients could promote the development of microbial resistance [14]. Furthermore, there is a marked and rapid tolerance to the gastrokinetic effects of both metoclopramide and erythromycin in critically ill patients [11]. Safer more effective gastrokinetic agents that have a sustained effect could improve nutrient delivery and thereby clinical outcomes in patients with critical illness.

Motilin is an endogenous peptide, produced mainly in the duodenum, whose physiological action is mediated by motilin receptors located on enteric neurons and on the smooth muscle of the gut. Endogenous motilin is responsible for the initiation of phase III contractions of the migrating motor complex (MMC) [15], which is thought to perform a “housekeeping role” by clearing the stomach and intestine of luminal secretions, undigested material and bacteria during the interdigestive period. Administration of exogenous drugs that stimulate motilin receptors accelerate gastric emptying [16].

Camicinal (GSK962040) is a first-in-class small molecule (non-macrolide) motilin receptor agonist with the potential to accelerate gastric emptying [17, 18]. Camicinal was designed to enhance the specificity for the recombinant human motilin receptor and potentially decrease the negative characteristics encountered with compounds with complex and nonspecific motilide structures. Camicinal has been shown to accelerate gastric emptying by 30–40 % in healthy volunteers as single (50–150 mg) or 14-day repeat oral doses (50–125 mg) [19, 20]. Similarly, in patients with type 1 diabetes and gastroparesis, camicinal accelerated gastric emptying by 35–60 % [21].

Enhancing GE in critically ill patients receiving EN may improve the delivery of nutrition and thereby improve nutritional and clinical outcomes. The objectives of this study were to assess the effects of single doses of camicinal on gastric emptying, by the ^13^C-octanoic acid breath test, acetaminophen absorption test, and glucose absorption by 3-O-methyl glucose (3-OMG) in critically ill patients who were “intolerant” to enteral feeding.

## Methods

The study was conducted in the intensive care unit (ICU) at the Royal Adelaide Hospital in Adelaide, South Australia, which is a mixed tertiary referral unit with a university affiliation. The study was run in accordance with “good clinical practice” (GCP), the Declaration of Helsinki and the National Health and Medical Research Council of Australia guidelines on research conducted on unconscious patients after obtaining informed consent from each patient’s next of kin. The study was approved by the Royal Adelaide Hospital Research Ethics Committee (Approval Number: 090924). The study protocol (GSK number 112571) was registered with the US NIH clinicaltrials.gov on 23 December 2009 (Identifier NCT01039805).

### Patients

Eligible patients were males and females between 18 and 85 years of age, undergoing invasive mechanical ventilation in the ICU who developed “feed intolerance” during nasogastric feeding. Patients were fed according to the Royal Adelaide Hospital feeding protocol, which is to commence 1 kcal/mL standard liquid nutrient feed at goal rate (to a maximum of 80 mL/h) [21], unless there are contraindications to enteral feed and to check GRVs every 6 hours [14]. “Feed intolerance” was defined as a single GRV ≥ 200 mL occurring at least 6 hours after commencing liquid nutrient at ≥ 40 kcal/hr [22]. Patients were expected to remain mechanically ventilated for at least 48 hours after enrollment and expected to survive for at least 24 hours after dosing of study medication. Additional inclusion criteria included the following: body weight ≥ 50 kg, average QTcB or QTcF < 450 msec; or QTc < 480 msec in subjects with bundle branch block, aspartate aminotransferase and alanine transaminase < 3 times the upper limit of normal; alkaline phosphatase and bilirubin ≤ twice the upper limit of normal. Patients were excluded if there was a known history of hepatitis B, C or HIV, they had received a drug known to have gastrokinetic effects in the previous 24 hours (e.g., erythromycin, azithromycin or metoclopramide), they had mechanical bowel obstruction, they were pregnant or lactating women, the investigator did not think they would be able to complete the study, they had a current or chronic history of liver disease, or known hepatic or biliary abnormalities (with the exception of Gilbert’s syndrome or asymptomatic gallstones), they had participated in a clinical trial and had received an investigational product within the following time period prior to the first dosing day in the current study: 30 days, five half-lives or twice the duration of the biological effect of the investigational product (whichever is longer), they were receiving or likely to receive drugs known to inhibit or induce CYP3A4 within the restricted timeframe relative to dosing of study medication, they had renal failure requiring replacement therapy (dialysis or filtration), the reason for admission to ICU was an overdose (deliberate or accidental; medicinal product or not), they had altered upper gastrointestinal tract anatomy, they had undergone upper gastrointestinal tract surgery on this admission to ICU, they had a gastric pacemaker, were receiving parenteral feeding, had sensitivity to any of the study medications, or components thereof.

All patients had a feeding tube in situ with the distal tip either 10 cm below the gastro-esophageal junction or clearly visualized in the stomach on plain abdominal radiograph.

### Study design

This trial was designed as a randomized, double-blind, parallel, single-dose, placebo-controlled study. It enrolled two cohorts of subjects. In the initial cohort, eligible patients were randomized in a ratio of 1:2 to receive a single dose of either placebo (*n* = 8) or 50 mg of camicinal (*n* = 15) in accordance with the randomization schedule generated by Discovery Biometrics GSK (GlaxoSmithKline, Harlow, UK), prior to the start of the study, using the validated internal software RandAll. Research staff accessed a web-based program to determine the allocation of each patient following consent. Hence allocation concealment was maintained. All patients, clinical and research staff at the site were blinded to the intervention. The dose of 50 mg was chosen as this dose had a proven gastrokinetic effect in healthy participants and was at the lower end of doses administered [19]. Because of the capacity for critically ill patients to have slow gastric emptying and a larger volume of distribution [23], a preplanned interim analysis of safety and pharmacokinetics (with investigators remaining blinded to pharmacodynamics) was performed and if the enteral dose of 50 mg appeared safe, but plasma concentrations of camicinal were not in the pharmacology range expected, the dose was increased and a second cohort of subjects received either placebo (*n* = 4) or 75 mg of camicinal (*n* = 6) to explore the pharmacodynamic effects of an increased dose.

Once enrolled, each subject completed “baseline”’ (pre-treatment) assessments of GE by ^13^C-octanoic acid breath test, acetaminophen absorption test, and glucose absorption test following 3-O-methyl glucose administration. Following the completion of the 4-hour gastric emptying measurement the patient was commenced on nutrient infusion at the rate they had been receiving prior to enrollment. On the following day after a 2-hour fast, study treatment was administered into the stomach via the NG tube as a 20-mL suspension in 90 mL of water or 110 mL of water at T = −90 min. Gastric emptying measurements were repeated 90 minutes after administration of randomized study treatment (i.e., T = 0 min). Subject safety was monitored via clinical observations, heart rate, and blood pressure measurements, electrocardiogram tracings and clinical laboratory determinations conducted from baseline to 5 days post-dose. A final follow-up evaluation was conducted between 7 and 10 days post-dose.

#### Test meal

Patients were studied in the supine position and the head of the bed was elevated to 30 °. Gastric contents were initially aspirated and discarded, and then 100 mL of liquid nutrient meal (Ensure; Abbott Australia, Macquarie Park, NSW, Australia), providing 106 kcal with 21 % of fat, was infused into the stomach over 5 minutes. The 100 mL feed included 100 mg of ^13^C-octanoate (100 mg/mL; Cambridge Isotope Laboratories, Inc., Andover, MA, USA), 1000 mg acetaminophen solution (Panadol™ syrup; GlaxoSmithKline, Ermington, NSW, Australia) and 3 g 3-O-methyl glucose (3-OMG; Sigma-Aldrich, Castle Hill, NSW, Australia). Time = 0 mins was defined as the time when all of the 100 mL of feed was infused into the stomach.

#### ^13^C-octanoic acid breath test

End-expiratory breath samples were obtained as previously described [24]. Samples were collected at baseline, every 5 minutes for the first hour, and every 15 minutes thereafter, for the subsequent 3 hours after meal administration. Breath samples were analyzed for CO_2_ concentration and the percentage of ^13^CO_2_ using an isotope ratio mass spectrometer (ABCA model 20 20, Europa Scientific, Crewe, UK). The values obtained were used to determine the percentage of ^13^C recovered per hour, which was plotted over time. These data were used to calculate the best fit curves for percent dose per hour and percent cumulative dose [9, 12]. From the resultant curves, the gastric half-emptying time (BTt½) was derived using the following formula [9, 12]: BTt½ = [−1/k] × ln[1 – 2-1/b]. The area under the recovery curve was used to calculate (i) gastric half-emptying time (BTt½), which is the time to 50 % of the total ^13^C recovered and (ii) the gastric emptying coefficient (GEC) as a global index for the gastric emptying rate (which accounts for the rate of appearance and disappearance of tracer in the breath) with the greater the number indicating the more rapid the emptying rate.

#### Blood samples

Blood samples were collected in chilled ethylenediaminetetraacetic acid (EDTA) tubes and separated within 30 minutes of collection for assessment of camicinal and acetaminophen concentrations. Blood was also collected into serum tubes for subsequent measurement of 3-OMG concentrations. Both serum and plasma were separated by centrifugation (3200 rpm for 15 minutes at 4 °C). Samples were then stored at −80 °C until assayed [25].

#### Glucose absorption (serum 3-O-methly glucose concentrations)

Glucose absorption was measured using 3-OMG, a previously validated technique in the critically ill [22, 26]. Arterial blood samples (5 mL) for plasma 3-OMG concentration were collected at baseline, 15, 30, 45, 60, 90, 120, and 240 minutes post-3-OMG dose for analysis by high-performance exchange chromatography. Peak 3-OMG concentration (Cmax), time to peak 3-OMG concentration (Tmax) and area under the curve between the end of the meal (T = 0) and 240 mins (AUC0–240) were calculated.

#### Plasma acetaminophen concentrations

Blood (5 mL) was obtained for measurement of plasma acetaminophen concentrations at 90, 60, and 30 minutes before the meal and 15, 30, 45, 60, 120 and 240 minutes after the meal, following acetaminophen dosing. Samples were analyzed by protein precipitation followed by liquid chromatography-tandem mass spectrometry (LC-MS/MS) analysis with a method range of 25–5000 ng/mL, using a 50 μL aliquot of human plasma. The concentration at 60 minutes after the end of the meal (C60) and area under the curve between the end of the meal (T = 0) and 60 minutes (AUC0–60) were calculated.

#### Plasma camicinal concentrations

Plasma camicinal concentrations were measured 90, 60, and 30 minutes before the meal and 15, 30, 45, 60, 120 and 240 minutes after the meal. Samples were analyzed using a validated HPLC with a method range 1 to 2000 ng/mL.

### Data analysis

#### Demographics

Sample size was estimated using data from a previous study where gastric emptying was measured using the ^13^C-octanoic acid breath test in an unselected group of 30 critically ill patients (BTt½ placebo mean estimate: 132.33 min, within-subject SD: 27.68 min) [27]. Based on these data it was estimated that a sample size of 12 would give 88 % power to detect a 30 % decrease of BTt½ (using a two-sided α = 0.05 level) between pre- and post-dose within each treatment group. Additional subjects were recruited because of expected dropouts between day 1 and day 2.

In this proof-of-principal study the acute effects of camicinal administered intragastrically were studied. Because absolute gastroparesis occurs in a proportion of critically ill patients [14, 28], it was anticipated that an enterally delivered drug may not empty adequately from the stomach and, therefore, would not be absorbed in patients with severe gastroparesis. For this reason it was decided to carry out an exploratory analysis in addition to the primary analysis in patients that achieved evaluable plasma camicinal concentrations.

#### Pharmacodynamics

Pharmacodynamic parameters were analyzed using a mixed model fitting treatment, visit (baseline or “pretreatment”, and posttreatment) and the interaction as fixed effects and subject as random. For each treatment, the point estimate and corresponding 95 % confidence interval for the difference “posttreatment – baseline” was constructed, using the residual error from the model. Where applicable, the log-transformed parameters were back transformed. If the confidence interval did not include zero (or one for log-transformed parameters), the difference was considered nominally significant at the 5 % level (unadjusted for multiple comparisons). All statistical analyses were carried out using SAS v. 9.2 (SAS Institute, Cary, NC, USA).

#### Pharmacokinetics

Plasma pharmacokinetic parameters for camicinal were derived using Phoenix WinNonlin v. 6.2 (Certara, Princeton, NJ, USA) from dried blood spot concentrations and are summarized as geometric means and coefficient of variation (CVb%) or median and range. Acetaminophen and 3-OMG plasma pharmacokinetic parameters were derived using Phoenix WinNonlin v. 6.2 from plasma concentrations and are summarized in Table 3 and 4.

#### Safety

An adverse event (AE) was defined as any untoward medical occurrence or unfavorable and unintended sign including an abnormal laboratory finding), symptom, or disease (new or exacerbated) temporally associated with the use of the study medication. Events that met the definition of an AE included: any abnormal laboratory test results (hematology, clinical chemistry, or urinalysis) or other safety assessments (e.g., ECGs, radiological scans, vital signs measurements), including those that worsen from baseline, and felt to be clinically significant in the medical and scientific judgment of the investigator, exacerbation of a chronic or intermittent pre-existing condition including either an increase in frequency and/or intensity of the condition, new conditions detected or diagnosed after study medication administration even though it may have been present prior to the start of the study, and/or signs, symptoms, or the clinical sequelae of a suspected interaction.

Using clinical judgment, the investigator assessed the relationship between study medication and the occurrence of each AE/serious adverse event (SAE) to determine study drug-related AEs.

## Results

**Fig. 1:**
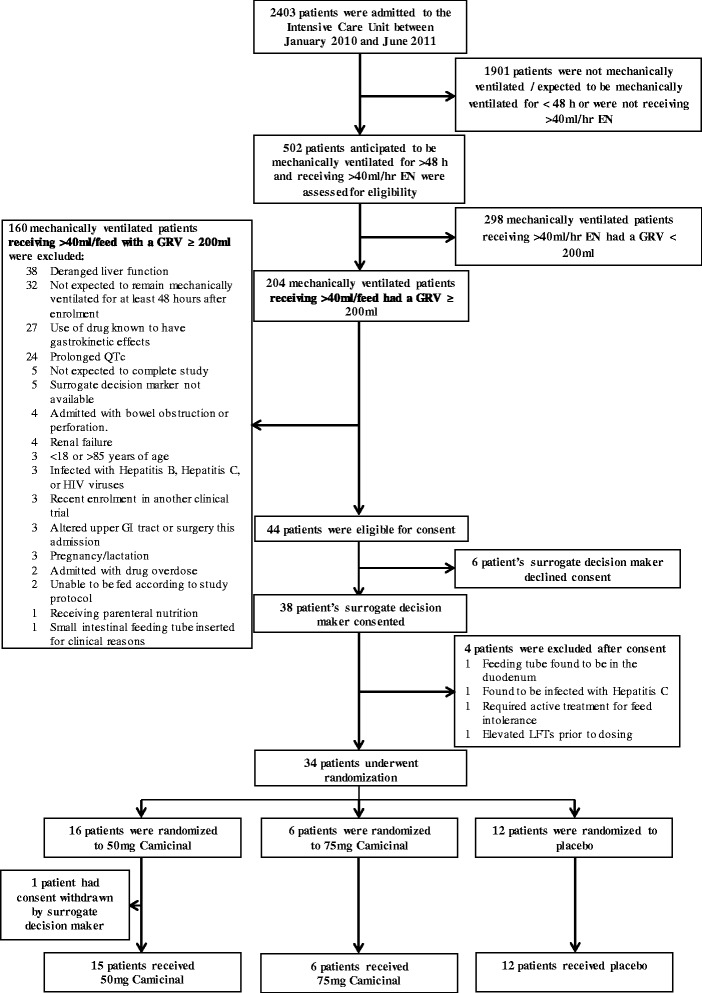
Consort diagram. Next of kin consent was provided for six patients who subsequently did not receive study drug because of the following: feeding tube found to be in the duodenum, found to be hepatitis C positive after consent given, consent given and subsequently withdrawn, patient withdrawn by investigator (not in best interest of patient to continue), liver function tests elevated meeting exclusion criteria, patient died after consent given but prior to receiving study treatment. *EN* enteral nutrition, *GRV* gastric residual volumes

Between January 2010 and June 2011, 204 patients were screened (Fig. 1). A total of 33 patients were randomized into the study with similar demographics across the treatment groups (Table 1).

**Table 1:** Patient demographics

		Camicinal	
	Placebo	50 mg	75 mg
	*N* = 12	*N* = 15	*N* = 6
Age in years, mean (SD)	46 (17)	63 (17)	42 (14)
Sex, n (%)			
Female:	2 (17)	6 (40)	1 (16)
Male:	10 (83)	9 (60)	5 (83)
BMI (kg/m^2^), mean (SD)	28.8 (5.8)	29.0(6.2)	28.7 (3.7)
Height (cm), mean (SD)	173 (8)	172 (10)	178 (9)
Weight (kg), mean (SD)	86 (17)	86 (20)	91 (17)
Baseline renal function			
Baseline creatinine (μmol/L), mean (SD)	73.1 (33)	89.3 (84)	56.0 (7.3)
Baseline hepatic function			
ALT (IU/L), mean (SD)	44.6 (30)	34.7 (22)	58.3 (39)
AST (IU/L), mean (SD)	43.2 (23)	35.6 (19)	55.2 (39)
Total bilirubin (μmol/L), mean (SD)	10.5 (15)	10.2 (13)	5.7 (3.7)
Admission category, n (%)			
Trauma	5 (42)	4 (27)	1 (17)
Head injury	1 (8)	3 (20)	1 (17)
Respiratory failure	1 (8)	3 (20)	0
Sepsis	2 (17)	2 (13)	0
Other	3 (25)	3 (20)	4 (67)
Illness severity and duration			
APACHE II, median (min, max)	17.5 (6, 37)	19 (5, 30)	14 (7, 29)
Days in ICU prior to enrollment, median (min, max)	4 (1, 9)	4 (2, 19)	4.5 (1, 7)
Days in ICU prior to study, median (min, max)	5 (2, 10)	5.5 (2, 9)	5 (2, 19)
Days in hospital prior to study, median (min, max)	5 (2, 10)	7 (3, 26)	5.5 (3, 9)
Concomitant chronic illnesses, n			
Diabetes	3	0	0
Concomitant medications, n			
Catecholamines	2	6	1
Opioids/opiates	12	14	4
Muscle relaxant	3	3	0
EN prior to enrollment			
Time from starting EN to development of feed intolerance; hours median (min, max)	29 (3, 162)	55 (5, 396)	50 (8, 81)
EN administered (EN delivered – discarded) in 24 hours prior to study eligibility; mL, median (min, max)	1130 (415, 1547)	950 (140, 1920)	904 (305, 1388)

*SD* standard deviation, *BMI* body mass index, *ALT* alanine transaminase, AST aspartate transaminase, *APACHE II*, Acute Physiologic Assessment and Chronic Health Evaluation II, *ICU* intensive care unit, EN enteral nutrition

### Plasma camicinal

Following intragastric administration, camicinal concentrations (AUC_0-24h_; geometric mean (%CV)) were 5205.9 (151.0) and 13,245.9 (42.0) ng.h/mL following 50 and 75 mg doses, respectively, with median Tmax 1.5 h (range 0.25, 7.5 h) and 1.25 (min: 1, max: 3 h). In two patients receiving 50 mg, plasma camicinal exposures did not increase within the pharmacodynamic evaluation period (the first 240 minutes post meal) with Tmax occurring in these subjects at 3 and 7.5 h, respectively.

In the 50 mg group, analyses were performed as “intention to treat”, which included all patients (*n* = 15), and a “modified per protocol analysis” including only those patients in whom the drug was emptied from the stomach and absorbed, thereby, resulting in detectable plasma concentrations of camicinal (*n* = 13).

### ^13^C-octanoic acid breath test

Pretreatment (baseline) gastric emptying was not similar across the different treatment groups. The pretreatment gastric emptying was slowest in the patients that subsequently received 50 mg and fastest in the patients that subsequently received 75 mg camicinal. The baseline geometric mean values for BTt½ were 56.87 (80.01 % between subject CV), 116.98 (98.37 % between subject CV), and 45.79 (77.48 % between subject CV) minutes for placebo, 50 mg and 75 mg camicinal, respectively. While placebo and 75 mg of camicinal had no effect on gastric emptying compared to baseline there was a strong trend to accelerated gastric emptying following the administration of 50 mg camicinal (Table 2). When analyzing data only from patients who achieved evaluable camicinal plasma levels during the GE assessment period, the acceleration of gastric emptying was more prominent (Table 2). Of the two patients who did not appear to absorb the drug during the period when gastric emptying was assessed (both received 50 mg camicinal), one had a fourfold increase in BTt½ from baseline (138.5 min to 585.1 min), indicating slowing of GE on the day the drug was administered and the other subject had no change from baseline (63.8 min to 68.8 min).

**Table 2:** Gastric emptying measured by ^13^C-octanoic acid breath test: baseline vs. post study drug comparison

^13^C-octanoic acid breath test parameters	Comparison	Baseline (mean)	Post study drug (mean)	Point estimate	95 % CI
BTt½ (min)^*^	50 mg (*n* = 15)	117	76	0.65	(0.39, 1.08)
	50 mg (*n* = 13)^#^	121	65	0.54	(0.32, 0.91)
	75 mg (*n* = 6)	46	85	1.85	(0.82, 4.15)
	Placebo (*n* = 12)	57	69	1.21	(0.68, 2.15)
GEC	50 mg (*n* = 15)	2.51	2.82	0.31	(−0.16, 0.77)
	50 mg (*n* = 13)^#^	2.51	3.05	0.55	(0.09, 1.00)
	75 mg (*n* = 6)	3.09	2.60	−0.49	(−1.23, 0.25)
	Placebo (*n* = 12)	2.90	2.83	−0.06	(−0.58, 0.46)

Least squares means and point estimates are geometric least squares means and ratios for log-transformed parameters. ^#^Two subjects administered 50 mg camicinal had low drug exposure. Analyses were performed with and without these subjects

*BTt½* breath test gastric time to half emptying, *GEC* gastric emptying coefficient

^*^Log-transformed

### Glucose absorption (3-OMG concentrations)

**Fig. 2:**
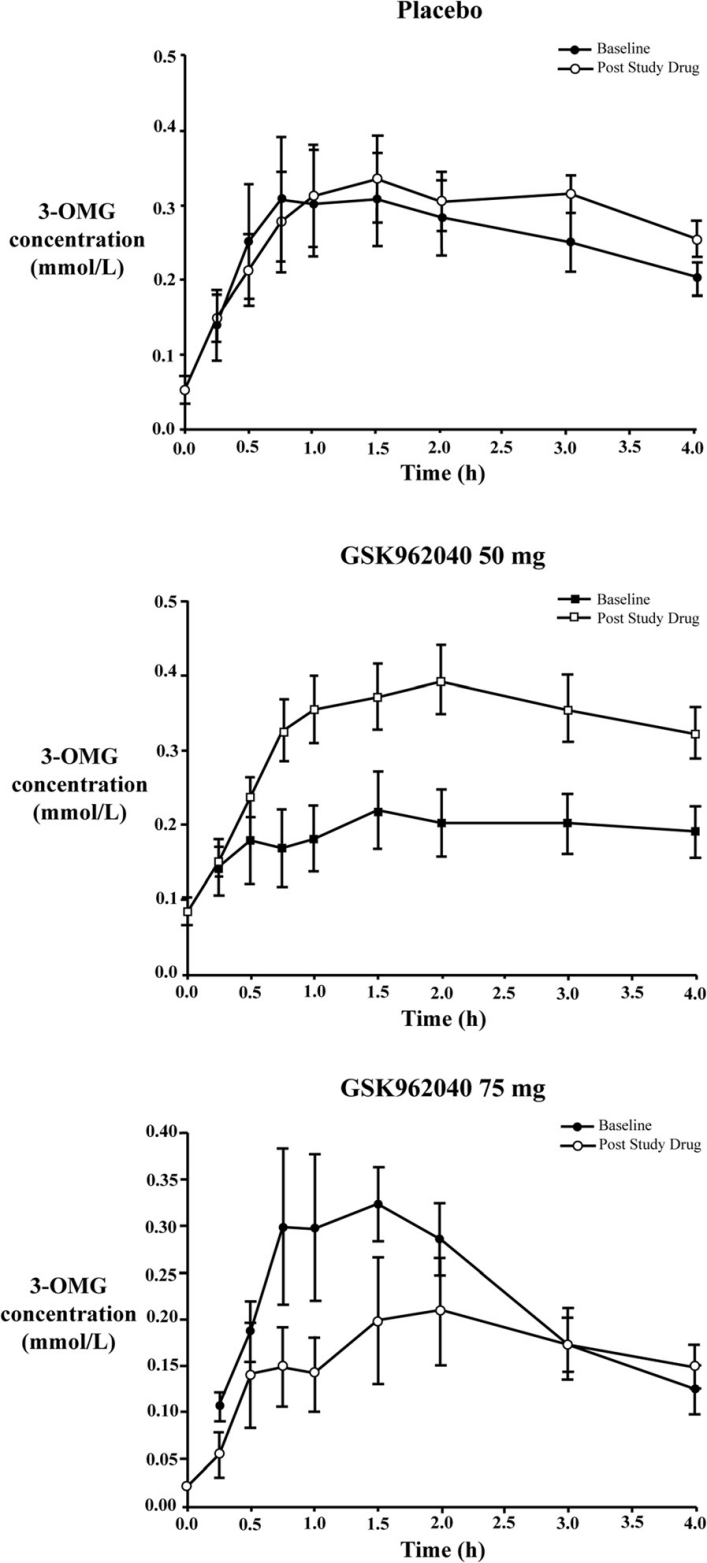
3-OMG (mean ± SEM) plasma concentrations vs. time in the placebo and 50 mg dose groups. *3-OMG* 3-O-methyl glucose

Pretreatment absorption was greater at baseline in both the placebo and the 75 mg arms compared to the 50 mg group. Camicinal 50 mg substantially increased glucose absorption in the first hour and for four hours after the meal (Fig. 2, Table 3).

**Table 3:** 3-O-methyl glucose absorption: post-dose vs. baseline comparison (excluding the *n* = 2 without adequate camicinal exposures)

Plasma 3-OMG	Comparison	Baseline	Post study drug	Point estimate	95 % CI
		(mean)	(mean)		
AUC(0–240) (mmol.min/L)^*^	50 mg (*n* = 15)	28.630	71.632	2.502	(1.683, 3.719)
	50 mg (*n* = 13)^#^	33.042	74.587	2.257	(1.478, 3.449)
	75 mg (*n* = 6)	48.373	35.048	0.725	(0.387, 1.356)
	Placebo (*n* = 12)	45.791	60.699	1.326	(0.851, 2.065)
AUC(0–60) (mmol.min/L)^*^	50 mg (*n* = 15)	2.962	11.646	3.932	(2.037, 7.588)
	50 mg (*n* = 13)^#^	3.779	12.390	3.279	(1.631, 6.592)
	75 mg (*n* = 6)	9.698	4.914	0.507	(0.179, 1.433)
	Placebo (*n* = 12)	5.935	7.464	1.258	(0.603, 2.623)
Cmax (mmol/L)^*^	50 mg (*n* = 15)	0.1905	0.4287	2.2505	(1.5736, 3.2186)
	50 mg (*n* = 13)^#^	0.2107	0.4496	2.1344	(1.4389, 3.1662)
	75 mg (*n* = 6)	0.3530	0.2512	0.7116	(0.4042, 1.2530)
	Placebo (*n* = 12)	0.3035	0.3824	1.2600	(0.8446, 1.8797)

*3-OMG* 3-O-methyl glucose, *CI* confidence interval, *AUC* area under the curve, *Cmax* peak 3-OMG concentration

^*^Log-transformed

^#^Two subjects administered 50 mg camicinal had low drug exposure. Analyses were performed with and without these subjects

### Plasma acetaminophen

There was a trend for the absorption, as demonstrated by increased exposure, of acetaminophen to increase after dosing with 50 mg dose (Table 4) compared to its absorption at baseline.

**Table 4:** Acetaminophen absorption: post-dose vs. baseline comparison (excluding the *n* = 2 without adequate camicinal exposures)

Plasma acetaminophen	Comparison	Baseline	Post study drug	Point estimate	95 % CI
		(mean)	(mean)		
C60 (ug/mL)^*^	50 mg (*n* = 15)	4.308	6.741	1.565	(0.962, 2.545)
	50 mg (*n* = 13)^#^	4.708	7.043	1.496	(0.874, 2.560)
	75 mg (*n* = 6)	5.398	6.315	1.170	(0.568, 2.409)
	Placebo (*n* = 12)	6.291	6.609	1.051	(0.630, 1.751)
AUC(_0–60_) (ug.min/mL)^*^	50 mg (*n* = 15)	159.31	256.93	1.61	(0.97, 2.67)
	50 mg (*n* = 13)^#^	169.80	293.52	1.73	(0.99, 3.03)
	75 mg (*n* = 6)	210.81	239.86	1.14	(0.54, 2.41)
	Placebo (*n* = 12)	224.21	230.08	1.03	(0.60, 1.74)

*CI* confidence interval, *AUC* area under the curve

^*^Log-transformed

^#^Two subjects administered 50 mg camicinal had low drug exposure. Analyses were performed with and without these subjects

### Safety

The frequency of reported AEs was similar across both doses of camicinal and placebo (Table 5). Serious adverse events (SAE) were reported for three subjects. There were two fatal SAEs, one event of severe sepsis and one event of brain herniation, both in the 50 mg arm. One subject in the placebo arm experienced nonfatal cardiac arrest. None of the SAEs were assessed as related to study treatment. The effect of camicinal on QTc was measured and no effect was found.

**Table 5:** Summary of adverse events occurring in ≥ 2 subjects

Adverse events:		Camicinal		
	Placebo	50 mg	75 mg	Total
	*n* = 12	*n* = 15	*n* = 6	*n* = 33
	*n* (%)	*n* (%)	*n* (%)	*n* (%)
No. subjects with any AE	9 (75)	12 (80)	5 (83)	26 (79)
Most frequent AEs (≥2 subjects in any single group)				
Gamma-glutamyltransferase increased	4 (33)	0	2 (33)	6 (18)
Oral candidiasis	0	2 (13)	1 (17)	3 (9)
Blood alkaline phosphatase increased	2 (17)	0	1 (17)	3 (9)
Decubitus ulcer	1 (8)	0	2 (33.)	3 (9)
Constipation	1 (8)	2 (13)	0	3 (9)
Vomiting	2 (17)	1 (7)	0	3 (9)
Diarrhea	2 (17)	0	0	2 (6)
Liver function test abnormal	0	2 (13)	0	2 (6)
Supraventricular tachycardia	0	2 (13)	0	2 (6)

*AE* adverse events

## Discussion

The objectives of this study were to evaluate the acute effects of camicinal in critically ill patients who were intolerant of EN. The most important findings from this study were that, when absorbed, a single dose of 50 mg camicinal accelerated gastric emptying and increased glucose absorption in patients with slow gastric emptying at baseline. In addition, while the drug was administered enterally, plasma concentrations reflecting pharmacological dosing occurred in the majority of patients. Adverse events were similar across all treatment arms.

Camicinal was designed using the recombinant human motilin receptor to enhance the specificity for the receptor and potentially decrease the negative characteristics encountered with compounds comprised of complex and nonspecific motilide structures [18]. This is the first study in which camicinal was administered to critically ill subjects with enteral feed intolerance.

Gastric delivery of enteral nutrition is frequently limited in the critically ill by slow gastric emptying, which occurs in up to 30–50 % of mechanically ventilated patients [3, 29]. This is commonly treated with gastrokinetic agents, the aim of which are to accelerate delivery of nutrients to the small intestine and augment nutrient absorption, thereby, improving nutritional and clinical outcomes. The gastrokinetic agents currently used are limited by side effects and are also subject to rapid tachyphylaxis [3, 30]. New drugs that persistently accelerate gastric emptying and have adequate safety profiles are needed for the treatment of slow gastric emptying in critical illness. This study shows that camicinal accelerates gastric emptying and that this results in augmented glucose absorption. Studies to evaluate the effects of multiple doses of camicinal in the critically ill are now warranted. Additional studies looking for a sustained effect on calorie delivery are also needed.

This is the first study to demonstrate that gastric emptying, when accelerated by a gastrokinetic drug, increases nutrient absorption. This is a predictable result as it has been previously demonstrated that there is a strong association between GE and glucose absorption in critical illness [31]. However, small intestinal absorption of both glucose and fat is impaired in critical illness independent of the rate of gastric emptying [32, 33], so the quantification of improvement in absorption following gastric acceleration is important. This study demonstrated that glucose absorption increased more than twofold following administration of 50 mg of camicinal, which is a magnitude of effect that has the capacity to be clinically relevant [32]. It has been previously demonstrated that erythromycin increases the absorption of glucose when nutrient is delivered into the small intestine but fat absorption may be reduced [22]. Octanoic acid, a medium-chain fatty acid, also showed augmented absorption when camicinal was administered. Further study on the effect of gastrokinetic drugs on nutrient absorption and clinical outcomes is warranted.

At present camicinal is only available as an enteral formulation. As gastric emptying can be substantially delayed in feed-intolerant critically ill patients [14], it was anticipated that emptying rates in some patients would be so slow that small intestinal absorption would be negligible in the acute period after a single dose. However, pharmacokinetic data indicate that, when gastric emptying occurred, mean concentrations of camicinal were comparable to healthy subjects and patients with diabetic gastroparesis [21]. This suggests that small intestinal absorption of the drug is unaffected by critical illness in most patients. We speculate that over an extended period of dosing some emptying would occur, even in patients with gastric stasis, which would allow subsequent doses of the drug to be absorbed more rapidly and allow for the pharmacologic effect to further enhance gastric emptying. Accordingly, a repeated dose study is warranted.

Three methods to evaluate gastric emptying were used in this study. All are indirect techniques that make use of a marker that is impermeable to gastric mucosa but is freely absorbed from the small intestine [24]. Each of the tests has advantages and limitations but the measurement of ^13^C-octanoic acid breath test, 3-OMG, and acetaminophen tests, which quantify lipid, glucose, and drug absorption respectively, is a strength of this study [28]. All demonstrated acceleration in gastric emptying following the absorption of 50 mg of camicinal although this was more marked using 3-OMG and the breath test. Acetaminophen absorption is a difficult test to interpret in the critically ill given the need to use acetaminophen for clinical purposes as well as variations in first pass effects due to hepatic metabolism. Differences in fat and glucose absorption may also account for some of the difference in results between 3-OMG and the ^13^C-octanoic acid breath test [22, 34].

Camicinal 50 mg, but not 75 mg, accelerated gastric emptying in the critically ill. The lack of effect observed following 75 mg may be due to a combination of the small sample size studied following this dose and the more normal (rather than slow) gastric emptying rate in this treatment arm at baseline. The normal baseline gastric emptying rate may well reflect that the cohort receiving this dose was younger and less severely ill than those who received 50 mg of camicinal [35]. Motilin agonists are unlikely to accelerate gastric emptying when the emptying rate at baseline is normal or rapid [36]. Previous data in healthy volunteers given camicinal show improvements in GE BTt½ with increased doses with a plateau at the greatest dose studied [19, 20]. The effect of erythromycin, a motilin receptor agonist, on gastrointestinal motility is dose-dependent, such that increasing doses may not accelerate gastric emptying as much as low or moderate doses [36, 37].

Interpretation of these data is limited by the pretreatment differences in gastric emptying, which occurred by chance. As the investigators remained blinded and randomization was not stratified according to baseline gastric emptying it happened that gastric emptying was slower in the group randomized to 50 mg camicinal. Because the gastrokinetic effect of drugs in the critically ill depend on baseline gastric emptying [25, 36], such that acceleration may only occur when baseline GE is slow, it is not possible to accurately determine from the results of this study as to whether 75 mg of camicinal could accelerate GE more than 50 mg and further dose-finding studies should be performed.

A further limitation is that the pretreatment gastric emptying was more rapid in those that received placebo. This was somewhat surprising given that patients were eligible only if GRV was ≥200 mL. While large GRV values are predictive of delayed gastric emptying [38, 39], effects on gastrointestinal motility may well vary with the severity of critical illness [40]. Indeed there is wide variation in the threshold GRV used to define feed intolerance, with volumes between 150 and 500 used to identify patients with slow gastric emptying [41–43], which limits generalizability of these data. Moreover, the time from measurement of the GRV until the measurement of gastric emptying may have been sufficient for some patients with slow gastric emptying to recover somewhat from their illness and for gastric emptying to improve [3]. This finding may support the practice by some groups of treating slow GE only after a second large GRV [44].

While pharmacokinetic data are presented, accurate assessment of half-life was not feasible in this study as PK samples were taken only up to 24 hours, while the half-life of the compound is about 30 hours. The short half-life estimates reported here should, therefore, be treated with caution. However, it appears that the elimination phase of subjects with critical illness parallels that in healthy volunteers with no evidence of changes in elimination of camicinal in this patient population [19]. Finally, the frequency of adverse events was similar for both doses of camicinal and placebo, with no treatment-related trends in clinical chemistry, vital signs, or electrocardiogram parameters. There were no serious adverse events assessed as related to study treatment in this small study and hence camicinal was generally safe and well tolerated in this critically ill population. Additional studies with more patients are needed to further characterize the safety profile of camicinal.

## Conclusions

In this phase II, proof-of-principal study a single dose of camicinal (50 mg or 75 mg) administered intragastrically to critically ill patients with feed intolerance was well tolerated. It was also well absorbed in almost all patients and, when absorbed, the 50 mg dose acutely accelerated gastric emptying resulting in improved nutrient absorption. Further clinical studies are required to determine the effect of camicinal on the success of gastric feeding and energy delivery in critical illness.

## Key messages

- A 50 g enteral dose of camicinal, when absorbed, accelerates gastric emptying and increases glucose absorption and emptying in feed-intolerant critically ill patients
- Further clinical evaluation with a larger patient cohort is needed to further characterize the safety profile of camicinal.

## Abbreviations

3-OMG, 3-O-methyl glucose; AV, adverse event; BT_t_½, gastric half-emptying time; Cmax, peak 3-OMG concentration; CVb%, coefficient of variation; EDTA, ethylenediaminetetraacetic acid; EN, enteral nutrition; GCP, good clinical practice; GE, gastric emptying; GEC, gastric emptying coefficient; GRV, gastric residual volumes; ICU, intensive care unit; LC-MS/MS, liquid chromatography-tandem mass spectrometry; MMC, migrating motor complex; SAE, serious adverse event; Tmax, time to peak 3-OMG concentration
